# Association between ADIPOQ +45T>G Polymorphism and Type 2 Diabetes: A Systematic Review and Meta-Analysis

**DOI:** 10.3390/ijms16010704

**Published:** 2014-12-30

**Authors:** Yaofu Fan, Kun Wang, Shuhang Xu, Guofang Chen, Hongjie Di, Meng Cao, Chao Liu

**Affiliations:** Endocrine and Diabetes Center, Jiangsu Province Hospital on Integration of Chinese and Western Medicine, Nanjing University of Chinese Medicine, Nanjing 210028, China; E-Mails: fanyaofu2010@163.com (Y.F.); wangkun112011@163.com (K.W.); xushuhang112011@163.com (S.X.); chenguofang112011@163.com (G.C.); dihongjie112011@163.com (H.D.); caomeng112011@163.com (M.C.)

**Keywords:** ADIPOQ, single nucleotide polymorphisms (SNPs), type 2 diabetes mellitus, meta-analysis

## Abstract

Recently, a number of studies have reported the association between the single nucleotide polymorphisms (SNPs) +45T>G polymorphism in the adiponectin (ADIPOQ) gene and type 2 diabetes mellitus (T2DM) risk, though the results are inconsistent. In order to obtain a more precise estimation of the relationship, a meta-analysis was performed. In this current study, the Medline, Embase, Pubmed, ISI Web of Knowledge, Ovid, Science Citation Index Expanded Database, Wanfang Database, and China National Knowledge Infrastructure were searched for eligible studies. Odds ratios (ORs) with 95% confidence intervals (CIs) were used to estimate the strength of association. Forty-five publications were included in the final meta-analysis with 9986 T2DM patients and 16,222 controls for ADIPOQ +45T>G polymorphism according to our inclusion and exclusion criteria. The +45T>G polymorphism was associated with an overall significantly increased risk of T2DM (G *vs.* T: OR = 1.18, 95% CI = 1.06–1.32; The dominant model: OR = 1.18, 95% CI = 1.03–1.33; The recessive model: OR = 1.47, 95% CI = 1.20–1.78; The homozygous model: OR = 1.62, 95% CI = 1.25–2.09; Except the heterozygous model: OR = 1.11, 95% CI = 0.98–1.24). Subgroup analysis revealed a significant association between the +45T>G polymorphism and T2D in an Asian population. Thus, this meta-analysis indicates that the G allele of the ADIPOQ +45T>G polymorphisms associated with a significantly increased risk of T2DM in the Asian population.

## 1. Introduction

Type 2 diabetes mellitus (T2DM) is one of the most common progressive metabolic diseases and poses a substantial burden on health-care systems globally. The International Diabetes Federation (IDF) has estimated that the prevalence of diabetes was 366 million worldwide, and is expected to increase up to 552 million by 2030 [1]. However, the mechanisms associated with T2DM remain uncertain. It is widely accepted that T2DM is a complex disease and both environmental and genetic factors can contribute to disease initiation as well as its evolution.

Adiponectin (encoded by ADIPOQ (also known as APM1, ACRP30 or GBP28)) is an important adipocytokine that is secreted by adipocytes and plays a key role in the inflammatory response that is associated with insulin-resistant states and T2DM [2,3,4]. The human *ADIPOQ* gene is mapped to chromosome 3q27 [5], displays a few polymorphisms in the promoter region (e.g., −11426A>G (rs16861194), −11391G>A (rs17300539), −11377C>G (rs267729)) or in the exon 2 (+45T>G, rs16861194) or in the intron2 (+276G>T, rs1501299), which could affect *ADIPOQ* gene transcription and its secretion [6]. In recent years, the associations of single nucleotide polymorphisms (SNPs) of the *ADIPOQ* gene with T2DM have been reported [7,8,9,10,11,12]. However, the results of these studies are still controversial, and show strong racial and regional variations. Therefore, we designed this meta-analysis synthesizing the data from single case-control studies to evaluate the genetic risk of the +45T>G polymorphism in the *ADIPOQ* gene for T2DM.

## 2. Results

### 2.1. Description of the Studies

**Figure 1:**
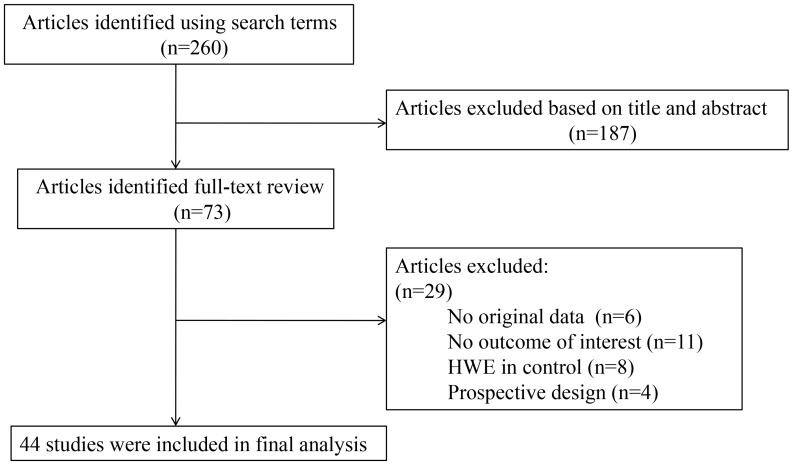
Study flow diagram.

A total of 260 studies were identified by the literature search. Of these, the first screening excluded 187 citations based on abstracts or titles, leaving 73 articles for full text reports. Applying the study inclusion criteria, 44 studies were included in this meta-analysis (Figure 1). A total of 9786 cases and 16,022 controls were included in the +45T>G analysis. Table 1 lists the main characteristics of the 44 studies eligible for the meta-analysis. These populations belong to Caucasian and Asian subgroups respectively. The Caucasian subgroup includes 14 individual studies and the Asian subgroup comprises 30 individual studies.

**Table 1:** Characteristics of studies included for investigation of associations between SNPs +45T>G and type 2 diabetes risk.

Study	Year	Country/Ethnicity	Study Design	Genotyping Method	Cases					Controls					HWE		
					TT	TG	GG	Allele G	Allele T	TT	TG	GG	Allele G	Allele T	χ^2^ Control Population	Cases	Controls
[6]	2009	Chinese/Asian	Population-based	ARMS-PCR	480	362	74	510	1322	483	389	98	585	1355	2.23	0.62	0.14
[12]	2010	Italy/Caucasian	Cohort	RT-PCR	370	117	16	149	857	359	126	18	162	844	2.68	0.08	0.1
[13]	2002	Japanese/Asian	Population-based	PCR-DS	164	169	51	271	497	251	183	46	275	685	2.18	0.48	0.14
[14]	2003	Japanese/Asian	Population-based	PCR-DS	78	66	20	106	222	90	74	15	104	254	＜0.01	0.31	0.97
[15]	2004	Chinese/Asian	Population-based	PCR-RFLP	104	71	20	111	279	98	74	15	104	270	0.04	0.14	0.84
[16]	2004	Chinese/Asian	Population-based	PCR-RFLP	8	46	24	94	62	39	35	11	57	113	0.49	0.04	0.48
[17]	2005	Korean/Asian	Hospital-based	SBE	252	202	39	280	706	201	181	45	271	583	0.2	0.87	0.65
[18]	2005	Chinese/Asian	Hospital-based	PCR-RFLP	56	36	12	60	148	48	38	4	46	134	1.08	0.11	0.3
[19]	2005	Chinese/Asian	Population-based	PCR-RFLP	53	46	16	78	152	46	44	5	54	136	1.82	0.25	0.18
[20]	2006	Chinese/Asian	Population-based	PCR-RFLP	103	69	23	115	275	78	57	4	65	213	2.9	0.04	0.09
[21]	2007	Chinese/Asian	Population-based	PCR-RFLP	36	19	2	23	91	75	16	3	22	166	2.92	0.79	0.09
[22]	2007	Chinese/Asian	Hospital-based	PCR-RFLP	67	36	17	70	170	60	45	15	75	165	1.94	＜0.01	0.16
[23]	2007	Chinese/Asian	Hospital-based	PCR-RFLP	80	92	28	148	252	122	72	6	84	316	1.44	0.85	0.23
[24]	2007	Chinese/Asian	Population-based	PCR-RFLP	39	48	13	74	126	58	40	3	46	156	1.6	0.77	0.21
[25]	2007	Chinese/Asian	Population-based	RT-PCR	20	94	54	202	134	68	60	22	104	196	2.05	0.03	0.15
[26]	2007	Chinese/Asian	Population-based	PCR-RFLP	89	79	12	103	257	152	114	20	154	418	0.05	0.32	0.83
[27]	2007	Chinese/Asian	Population-based	RT-PCR	90	36	12	60	216	48	64	20	104	160	0.03	＜0.01	0.86
[28]	2008	Chinese/Asian	Hospital-based	PCR–RFLP	134	135	20	175	403	59	38	6	50	156	＜0.01	0.07	0.97
[29]	2008	Chinese/Asian	Population-based	PCR-RFLP	126	115	14	143	367	76	40	4	48	192	0.21	0.06	0.65
[30]	2008	Chinese/Asian	Hospital-based	PCR-RFLP	103	75	17	109	281	79	53	6	65	211	0.61	0.53	0.43
[31]	2008	Chinese/Asian	Population-based	PCR-RFLP	55	26	16	58	136	53	41	4	49	147	1.31	＜0.01	0.25
[32]	2008	Chinese/Asian	Population-based	PCR-RFLP	167	123	22	167	457	85	75	7	89	245	3.7	0.92	0.05
[33]	2009	Chinese/Asian	Population-based	PCR-RFLP	44	44	18	80	132	28	24	6	36	80	0.06	0.23	0.8
[34]	2009	Chinese/Asian	Population-based	PCR-RFLP	71	44	11	66	186	47	54	11	76	148	0.64	0.28	0.42
[35]	2009	Chinese/Asian	Population-based	PCR-RFLP	68	52	11	74	188	59	42	4	50	160	1.1	0.81	0.29
[36]	2010	Chinese/Asian	Hospital-based	PCR-RFLP	38	47	15	77	123	60	37	3	43	157	0.92	0.94	0.34
[37]	2011	Chinese/Asian	Population-based	PCR-RFLP	209	99	19	137	517	206	103	20	143	515	2.09	0.12	0.15
[38]	2012	Chinese/Asian	Population-based	PCR-RFLP	88	54	11	76	230	88	62	8	78	238	0.48	0.5	0.49
[39]	2012	Chinese/Asian	Population-based	PCR-RFLP	97	46	4	54	240	135	52	2	56	322	1.53	0.6	0.22
[40]	2012	Chinese/Asian	Hospital-based	PCR-SSCP	114	134	26	186	362	84	50	7	64	218	0.02	0.13	0.9
[41]	2013	Chinese/Asian	Hospital-based	PCR-DS	75	79	26	131	229	64	48	8	64	176	0.06	0.49	0.8
[42]	2002	Italy/Caucasian	Hospital-based	ARMS-PCR	242	61	7	75	545	220	75	9	93	515	0.7	0.19	0.4
[43]	2004	French/Caucasian	Population-based	RT-PCR	24	6	1	8	54	2816	847	56	959	6479	0.72	0.44	0.4
[44]	2005	Finland/Caucasian	Cohort	-	235	23	0	23	493	255	26	2	30	536	2.04	0.45	0.15
[45]	2005	Spain/Caucasian	Population-based	PCR-SSCP	35	24	2	28	94	346	166	18	202	858	0.12	0.38	0.73
[46]	2006	Mexico/-	Population-based	PCR-RFLP	262	123	11	145	647	582	261	30	321	1425	0.01	0.44	0.91
[47]	2006	German/Caucasian	Cohort	RT-PCR	299	60	6	72	658	263	53	7	67	579	4.45	0.15	0.03
[48]	2007	UK/Caucasian	Population-based	PCR-RFLP	116	25	7	39	257	1968	536	35	606	4472	0.05	＜0.01	0.83
[49]	2008	Polish/Caucasian	Population-based	PCR-RFLP	117	10	2	14	244	108	8	1	10	224	3.16	＜0.01	0.08
[50]	2009	Russian/Caucasian	Population-based	PCR–RFLP	427	67	1	69	921	368	66	1	68	802	1.21	0.33	0.27
[51]	2009	Iranian/Caucasian	Population-based	PCR-RFLP	31	17	4	25	79	42	10	0	10	94	0.59	0.5	0.44
[52]	2010	Brazilian/Asian	Population-based	PCR-DS	93	95	12	119	281	100	85	15	115	285	0.28	0.05	0.6
[53]	2010	Iranian/Caucasian	Population-based	PCR-RFLP	171	63	7	77	405	117	47	9	65	281	2.08	0.68	0.15
[54]	2012	Saudi Arabia/ Caucasian	Population-based	ARMS-PCR	220	72	6	84	512	209	80	9	98	498	0.16	0.96	0.69

PCR, polymerase chain reaction; DS, direct sequencing; RFLP, restriction fragment length polymorphisms; RT, Real-Time; SBE, single base extension; ARMS, amplification refractory mutation system; SSCP, single strand conformation polymorphism; and HWE, Hardy-Weinberg equilibrium.

### 2.2. Pooled Analyses

In the whole population, a significant association was found between the *ADIPOQ* gene +45T>G polymorphism and the T2DM under allelic (OR: 1.18, 95% CI: 1.06–1.32, *p* = 0.002), dominant (OR: 1.18, 95% CI: 1.03–1.34, *p* = 0.014), recessive (OR: 1.47, 95% CI: 1.20–1.80, *p* < 0.001), homozygous (OR: 1.62, 95% CI: 1.25–2.09, *p* < 0.001), and heterozygous (OR: 1.11, 95% CI: 0.98–1.24, *p* = 0.11).

**Figure 2:**
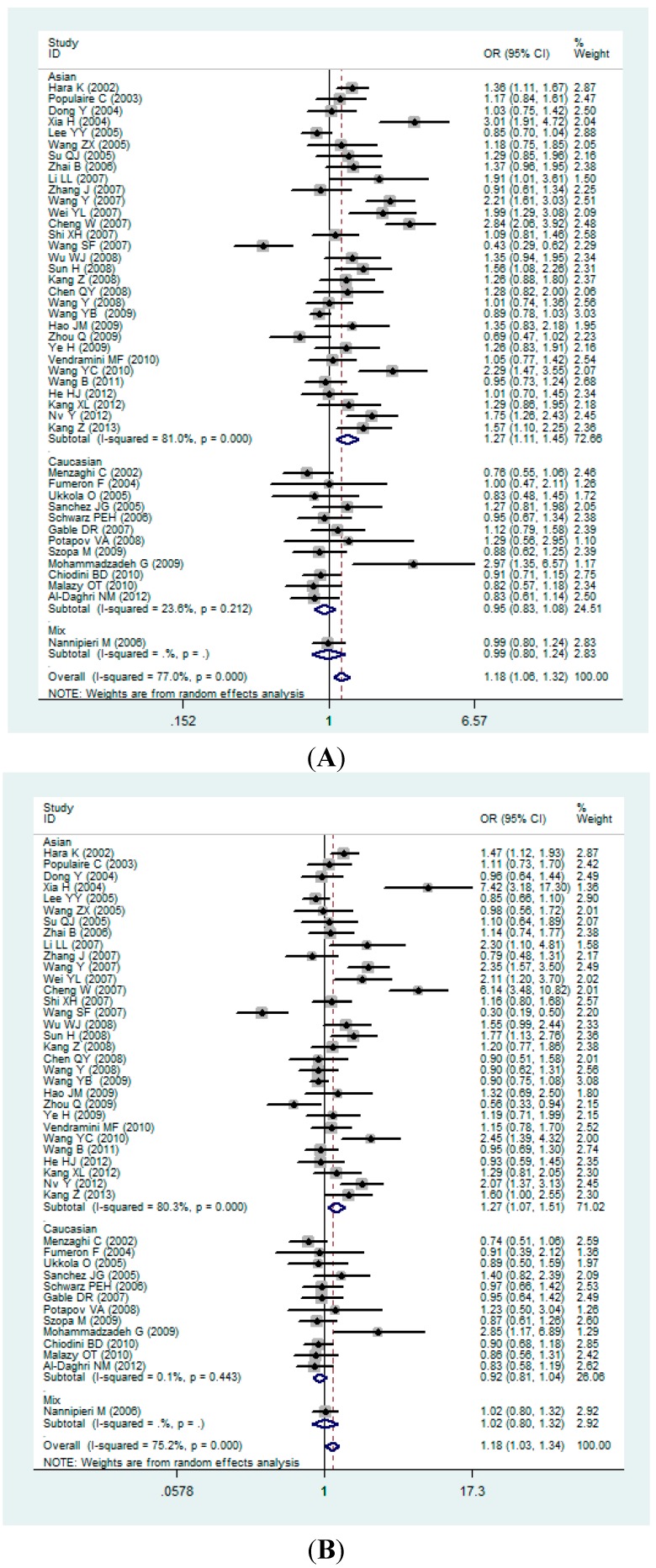

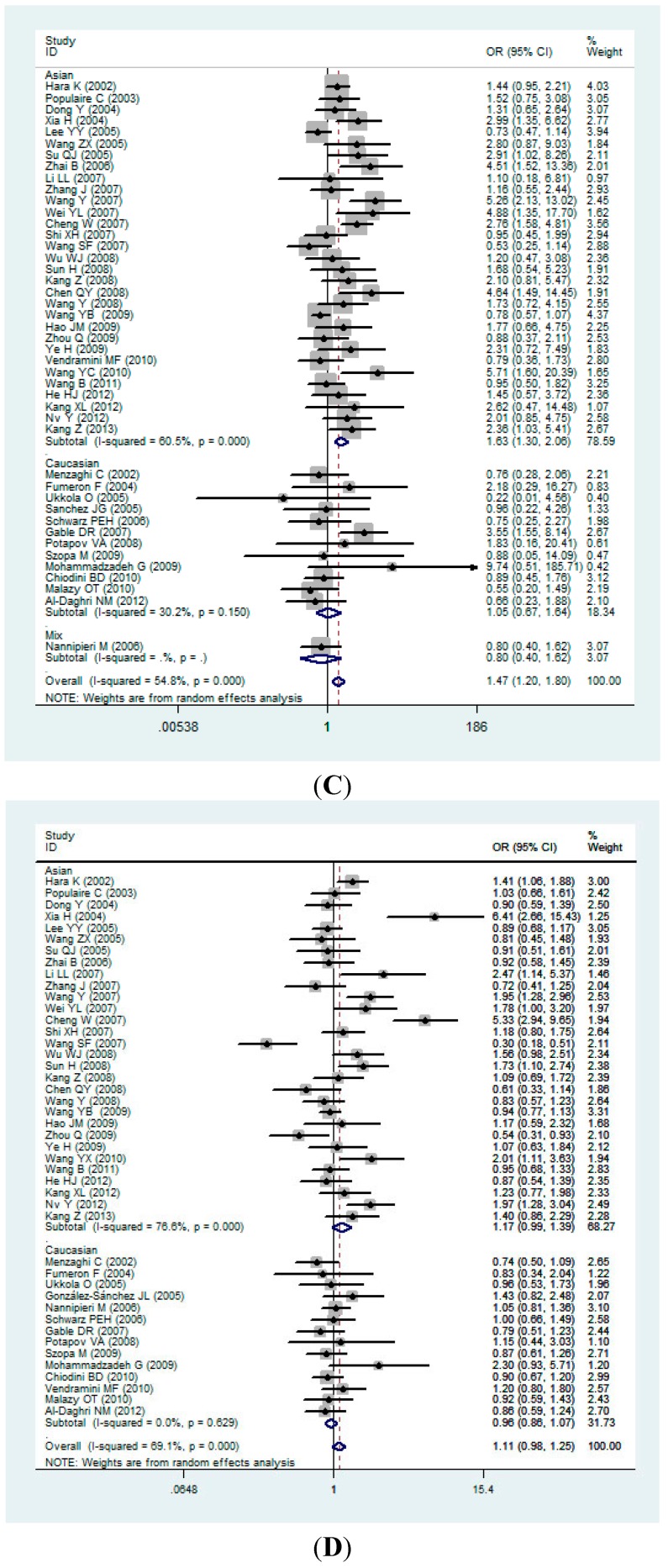

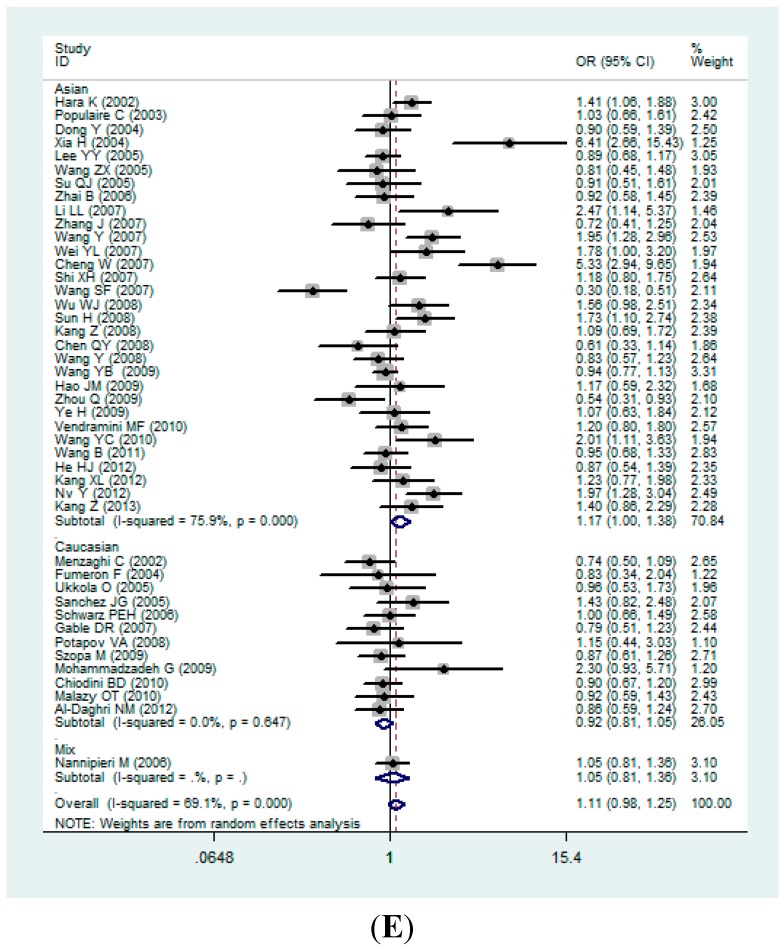
Forest plots for the *ADIPOQ* gene +45T>G polymorphism and T2DM risk in different genetic models. (**A**) Allelic model: G *vs.* T; (**B**) Dominant model: GG + GT *vs.* TT; (**C**) Recessive model: GG *vs.* GT + TT; (**D**) Homozygous model: GG *vs.* TT; and (**E**) Heterozygous model: GT *vs.* TT.

In the subgroup analysis, there was a significant association between them in the Asian population under allelic (OR: 1.27, 95% CI: 1.11–1.45, *p* < 0.001), dominant (OR: 1.27, 95% CI: 1.07–1.51, *p* = 0.007), recessive (OR: 1.63, 95% CI: 1.30–2.06, *p* < 0.001), homozygous (OR: 1.87, 95% CI: 1.38–2.54, *p* < 0.001), and heterozygous (OR: 1.17, 95% CI: 1.00–1.38, *p* < 0.001). However, in the Caucasian subgroup, there was no significant association between the *ADIPOQ* gene +45T>G polymorphism and T2DM under allelic (OR: 0.95, 95% CI: 0.83–1.08, *p* = 0.212), dominant (OR: 0.92, 95% CI: 0.81–1.04, *p* = 0.443), recessive (OR: 1.05, 95% CI: 0.67–1.64, *p* = 0.150), homozygous (OR: 1.03, 95% CI: 0.66–1.62, *p* = 0.145), and heterozygous (OR: 0.92, 95% CI: 0.81–1.05, *p* = 0.647). (Figure 2A–E).

### 2.3. Sensitivity Analysis

We first performed a sensitivity analysis by sequence, excluding individual studies to reflect the influence of the individual data set to the pooled ORs. The results showed that none of the individual studies influenced the final conclusion. The corresponding pooled Ors were not altered substantially (data not shown), indicating that our meta-analysis had reliable and stable results.

### 2.4. Publication Bias Analysis

**Figure 3:**
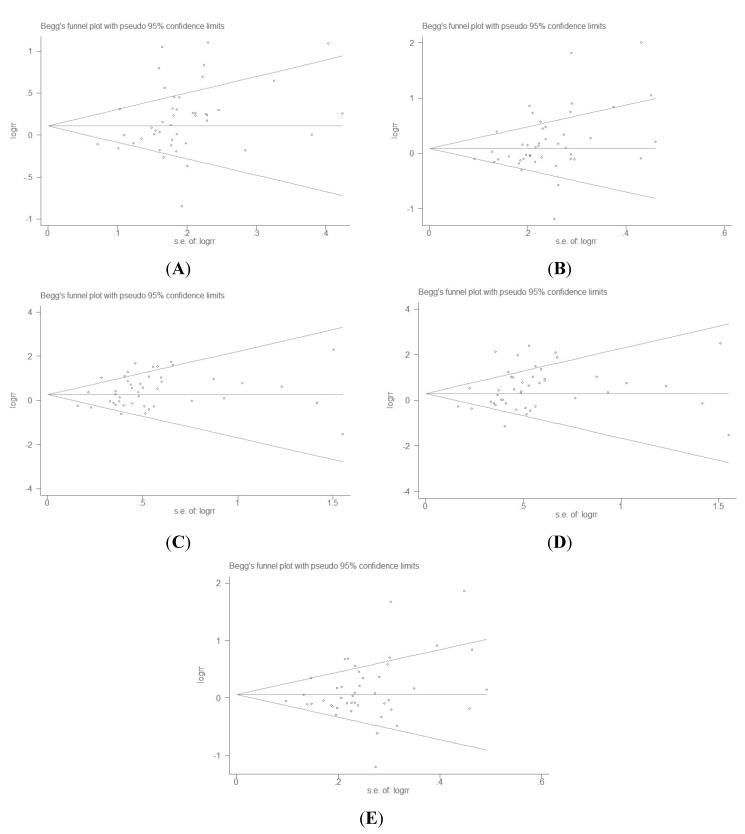
Funnel plots for *ADIPOQ* gene +45T>G polymorphism and T2DM risk in different genetic model. (**A**) Allelic model: G* vs.* T; (**B**) Dominant model: GG+GT* vs.* TT; (**C**) Recessive model: GG *vs.* GT+TT; (**D**) Homozygous model: GG* vs.* TT; and (**E**) Heterozygous model: GT* vs.* TT.

Begg’s funnel plot and Egger’s test were performed to determine whether the literature showed a publication bias. Begg’s funnel plots did not exist in overall comparisons except the allelic model and the dominant model (shown in Figure 3A–E), the effect sizes were asymmetrically distributed with publication bias visually present. In addition, the results of Egger’s regression test also showed evidence of publication bias (*p* = 0.023 for allele model, *p* = 0.016 for dominant model, *p* = 0.014 for recessive model, *p* = 0.019 for homozygous model, *p* = 0.094 for heterozygous model, respectively).

## 3. Discussion

The *ADIPOQ* gene is located on human chromosome 3q27, where a region composed of three exons that span 17 kb, identified as a susceptibility locus for metabolic syndrome and T2DM, has been reported [55,56,57]. T2DM is a complex heterogeneous group of metabolic disorders including hyperglycemia and impaired insulin action and/or insulin secretion, and a detailed etiology underlying T2DM is still unclear [58,59]. Therefore, it is necessary to identify the pathogenesis of T2DM. Recently, the *ADIPOQ* gene +45T>G polymorphism has been suggested to be implicated in the risk for type 2 diabetes, however, association studies have reported conflicting results. This may be due to a small sample size in each of the published studies and the ethnicity of these study populations. However, the results of these studies were still controversial. Therefore, we designed this meta-analysis to derive a more precise association between the *ADIPOQ* gene +45T>G polymorphism and T2DM risk.

In the current meta-analysis, a significant association was detected in the whole population between the *ADIPOQ* gene +45T>G polymorphism and T2DM under allelic, dominant, recessive and the homozygous genetic model, except the heterozygous model. Because the risk allele frequency of +45T>G differed greatly among ethnicities, we performed subgroup analyses according to ethnicity. We obtained the following results: In Asians, the *ADIPOQ* gene +45T>G polymorphism was significantly associated with risk of type 2 diabetes, while it was not found to be associated with risk of type 2 diabetes in Caucasians. In conclusion, it was indicated that the G allele of *ADIPOQ* gene +45T>G polymorphism might be a predisposing factor to T2DM in the Asian population, whereas we did not find any association between T2DM and *ADIPOQ* +45T>G in the Caucasian population. The discrepancy might be caused by differences in the *ADIPOQ* +45T>G genotype distribution in different ethnic backgrounds [60]. This result differs from that of Han *et al.* [61], who showed that the presence of +45T>G appeared to have no effect in Asians and whites. Menzaghi *et al.*, conducted a meta-analysis to explore the associations of different *ADIPOQ* SNPs with insulin resistance, T2DM and cardiovascular disease [62]. However, Menzaghi and colleagues did not observe significant global effects between +45T>G polymorphism considered in their meta-analysis. Li *et al.*, also observed no association between +45T>G polymorphism and T2DM on the Han Chinese population [63]. Their conclusions were not consistent with our study, and we found that the G *vs.* T allele of +45T>G might be associated with T2DM risk. One possible explanation is that different populations may have experienced very diverse environmental impacts during their evolution. In addition, different life style as well as study sample size might also have contributed to this difference.

Significant between-study heterogeneity existed in all models, which may affect the results of the present meta-analysis. Common sources of heterogeneity may be attributed to the diversity in ethnicity, sample size, genotype errors, publication bias and different study design (population-based, hospital-based, or cohort) and diagnostic criteria *etc.* [63,64,65]. We tried to clarify the sources of heterogeneity according to the subgroup analysis by ethnicity. However, we did not effectively remove the heterogeneity. We therefore assumed that all the potential sources mentioned above should be taken into account.

When explaining our results, some limitations of this meta-analysis should be considered. Several possible reasons may account for the difference between Asians and Caucasians in the association of the *ADIPOQ* gene +45T>G polymorphism with type 2 diabetes. Firstly, different genetic background may play a role. T2DM is the result of diverse gene-environment interactions; we could not retrieve more detailed individual data, which were available, such as occupation, histological types and so on. In the Asian subgroup, the result showed a high level of between-study heterogeneity, suggesting that the studies do not estimate the same effect due to different degree of bias. In addition, there are significant differences regarding etiological profiles between high and low incidence areas within Asian. Secondly, our search was limited to published English and Chinese language studies, with studies published in other languages systematically excluded. This may explain some publication bias in our meta-analysis, which may have affected the results of this meta-analysis in as far as those studies that had produced negative results might not have been published. Thirdly, type 2 diabetes is a complex disease which is affected by an interplay between many factors, including environmental exposure, life style, socioeconomic status and individual susceptibility [66,67]. It is possible that individual susceptibility in different ethnic groups may be modified by environmental exposure, life style and socioeconomic status in a different way. However, it is noted that although this meta-analysis has revealed a positive association between the *ADIPOQ* gene +45T>G polymorphism and type 2 diabetes, the result needs to be interpreted in cautiously. The total sample size of Asians in this meta-analysis is still relatively small (T2DM, 6299; Controls, 5673), which may restrict the statistical power for achieving a definitive conclusion. Therefore, case-control studies in larger samples are needed to confirm this correlation.

Some studies have demonstrated that the main insulin-sensitizing action of adiponectin results from decrease in hepatic gluconeogenesis, increase in muscle glucose transport and enhancement of energy consumption and fatty acid oxidation in peripheral tissues with the aim of increasing ATP production [68,69]. In addition, a few articles had reported that the potential role of adiponectin on insulin secretion, as well as on energy expenditure, through central action [70,71,72]. Fasshauer reported that T2DM is characterized by low-grade inflammation and increased circulating concentrations of inflammatory cytokines, which in turn are putative negative regulatory factors of the adiponectin gene [73]. Clinical and experimental animal studies also reported a decline in adiponectin levels seems to identify insulin resistance before the development of overt diabetes. Reduced adiponectin levels may play an important causal role in the development of insulin resistance and type 2 diabetes. In this sense, the progress of adiponectin analogues holds great promise for clinical use in the prevention and treatment of diabetes [74]. However, many questions need to be addressed before adiponectin can be used as a potent therapeutic target.

## 4. Materials and Methods

### 4.1. Literature Search Strategy

Potential eligible studies were identified by systematically searching the Medline, Embase, Pubmed, ISI Web of Knowledge, Ovid, Science Citation Index Expanded Database, Wanfang Database and China National Knowledge Infrastructure up to November 2013. The following free-text word or subject headings (Medical Subject Headings (MeSH)) terms were used: “diabetes mellitus” or “type 2 diabetes” or “type 2 diabetes mellitus” or “T2DM”, “adiponectin” or “adiponectin” or “APM1” or “ACDC” or “ADIPOQ”, “polymorphism” or “single nucleotide” or “single nucleotide polymorphisms” or “variant” or “mutation” or “mutant” or “SNP”. Two independent reviewers performed searches in duplicate. All eligible studies were retrieved, and their bibliographies were checked for other relevant publications. Only published studies with full-text articles were included in our meta-analysis.

### 4.2. Inclusion and Exclusion Criteria

We reviewed abstracts of all citations and retrieved studies. The following criteria were used to include published studies: (1) Published in English or in Chinese; (2) Case-control study or cohort study; (3) Supplied the available genotype frequencies in cases and controls; (4) Available data to estimate an odds ratio (OR) with 95% confidence interval (CI); and (5) Genotype distribution in controls were in Hardy-Weinberg equilibrium (HWE). Studies were excluded if one of the following existed: (1) No control group; (2) Studies that contained overlapping data; and (3) No report about the genotype frequency, or insufficient information for data extraction.

### 4.3. Data Extraction

Two investigators (Yaofu Fan and Kun Wang) extracted information from all eligible publications independently according to the inclusion criteria listed earlier. Any disagreement was adjudicated by consensus and consulting a third author (Shuhang Xu). The following characteristics were collected from each study: first author, year of publication, country/region, diagnosis criteria for T2DM, genotyping methods, major variant allele frequency in cases and controls, and *p* values for Hardy–Weinberg equilibrium (HWE) in the T2DM and control groups were summarized.

### 4.4. Statistical Methods

Odds ratios with 95% CIs were calculated to assess the associations between *ADIPOQ* +45T>G polymorphism and T2DM risk. Association under four different types of Ors was estimated, including homozygote model (GG *vs.* TT), heterozygous model (GT *vs.* TT), dominant model (GG + TG *vs.* TT), recessive model (GG *vs.* TG + TT) and an allele contrast model (G *vs.* T) for *ADIPOQ* +45T>G polymorphism, respectively. All meta-analysis were performed using Stata statistical software (STATA version SE-10.1; Stata Corporation, College Station, TX, USA). The HWE was checked by applying a Chi-square goodness-of-fit test. The heterogeneity among the studies was calculated by Chi-square-based *Q*-tests with significance set at *p* < 0.05 [75]. If the heterogeneity existed among the individual studies, the pooled OR was assessed using random-effect model (DerSimonian and Laird method) [76]. Or else, the fixed-effect model was adopted (the Mantel–Haenszel method) [77]. The *Z*-test was used to estimate the pooled OR and the significance level was set at *p* < 0.05. The significance of the pooled OR was determined by the *Z*-test, in which *p* < 0.05 was considered significant. A sensitivity analysis was performed to identify potential outliers. Funnel plots, Egger’s test and Begg’s test were used to evaluate publication bias.

## 5. Conclusions

This meta-analysis suggests that the *ADIPOQ* gene +45T>G polymorphism may be associated with susceptibility to T2DM in the Asian population. However, it is still necessary to conduct larger sample studies using standardized unbiased genotyping methods and to explore the association among different ethnicities in the future.
